# From Research to Practice: The Future of Cardiovascular Care

**DOI:** 10.7759/cureus.84473

**Published:** 2025-05-20

**Authors:** Prabhash C Manoria

**Affiliations:** 1 Cardiology, Director of Manoria Heart and Critical Care Hospital, Bhopal, IND

**Keywords:** artificial intelligence, cardiovascular disease, genomics, stem cells, wearables

## Abstract

Cardiovascular diseases (CVDs) remain the leading cause of mortality worldwide, necessitating continuous advancements in research and clinical practice. Research advancements have significantly transformed our understanding and management of CVDs. Recent breakthroughs in genomics, artificial intelligence (AI), wearable technology, and regenerative medicine are reshaping cardiovascular (CV) care. This review explores key innovations that bridge the gap between research and clinical application, emphasizing precision medicine, early detection, and personalized therapies. We also discuss challenges in implementation and future directions for optimizing CV outcomes.

## Introduction and background

Cardiovascular diseases (CVDs) remain the leading global cause of morbidity and mortality, accounting for nearly 18 million deaths annually and imposing a significant economic burden on healthcare systems worldwide [1,2]. Despite advancements in prevention, diagnosis, and treatment, such as statins, stent technology, and improved hypertension management, CVDs continue to challenge clinicians and researchers. The gap between scientific discovery and real-world clinical application persists, often delaying the adoption of breakthrough therapies that could save lives.

The future of cardiovascular (CV) care lies in bridging this translational gap, leveraging cutting-edge innovations such as precision medicine, artificial intelligence (AI), wearable technologies, and regenerative therapies. These advancements promise to revolutionize how we predict, diagnose, and treat heart disease, shifting from a reactive to a proactive and personalized approach [3]. For instance, AI-driven diagnostic devices like smartwatches [4] can detect early signs of heart failure (HF) before symptoms manifest, while gene-editing technologies like clustered regularly interspaced short palindromic repeats (CRISPR) may one day correct inherited cardiac disorders. AI/machine learning (ML) applications in echocardiography have also been used for automated disease detection in patients [5]. Wearable devices like smart wristbands, patches, fingerings, smart shirt, smartwatches, etc. are one of the non-invasive and cost-effective gadget aids in monitoring the risk of heart condition, which enable continuous remote monitoring, reducing hospital readmissions and empowering patients in their own care [4,6]. The WATCH AF Trial demonstrated that detecting atrial fibrillation using a commercially available smartwatch is feasible, highly accurate, widely applicable, convenient, and potentially cost-effective for large-scale screening or extended monitoring in high-risk individuals [7].

However, integrating these innovations into clinical practice presents challenges, including regulatory hurdles, cost barriers, data privacy concerns, and the need for physician training. Addressing these obstacles will require collaboration among researchers, clinicians, policymakers, and industry leaders to ensure equitable and scalable implementation.

This review explores the most promising research-driven advancements in cardiovascular medicine and their pathway to clinical adoption. By examining the latest developments in genomics, digital health, regenerative medicine, and minimally invasive interventions, we aim to highlight how these innovations can transform patient outcomes, ultimately shaping a future where cardiovascular care is more precise, preventive, and personalized than ever before.

## Review

Advances in CV diagnostics

1. Precision Medicine and Genomics

The integration of genomics into CV medicine has enabled personalized treatment approaches. Precision medicine, sometimes known as "personalized medicine" is an innovative approach to tailoring disease prevention and treatment that takes into account differences in people's genes, environments, and lifestyles, which aims to target the right treatments to the right patients at the right time [8]. Precision medicine has revolutionized CV care by tailoring treatments to individuals' genetic and molecular profiles. Advances in genomic sequencing, polygenic risk scores, and personalized medicine approaches have enhanced early detection, prevention, and targeted interventions for CVD [9,10]. 

Genomic studies have identified specific gene mutations associated with inherited CV conditions such as hypertrophic cardiomyopathy, long QT syndrome, and familial hypercholesterolemia. Early identification of these genetic predispositions enables proactive management through lifestyle modifications, pharmacologic interventions, and risk-reducing procedures.

Pharmacogenomics has also been crucial in optimizing CV drug therapy by assessing genetic variations that influence drug metabolism and efficacy [11]. For instance, variations in the CYP2C19 gene affect the response to antiplatelet therapy with clopidogrel, influencing treatment decisions for patients undergoing percutaneous coronary intervention (PCI) [12]. Similarly, genetic markers for warfarin metabolism guide individualized dosing to minimize the risk of bleeding complications [13]. The advent of gene-editing technologies such as CRISPR-Cas9 has opened new avenues for potential curative treatments of genetic CV disorders [14]. Ongoing research explores the possibility of correcting pathogenic mutations associated with conditions like Marfan syndrome [15] and familial hypercholesterolemia [16] at the molecular level.

Incorporating multi-omics approaches, including transcriptomics, proteomics, and metabolomics, further refines precision medicine strategies by providing a comprehensive understanding of disease mechanisms. These advancements enable more accurate risk stratification, early interventions, and the development of novel targeted therapies tailored to an individual's genetic and molecular profile.

2. AI and Digital Health

The AI and digital health technologies are transforming CV care by enhancing diagnostic accuracy, optimizing treatment strategies, and facilitating remote monitoring of patients [17]. The AI-driven algorithms, ML models, and wearable health devices are enabling real-time data collection and predictive analytics, thereby improving early detection and personalized treatment approaches [18]. Additionally, the AI models have revolutionized predictive tools in CVD due to their powerful data-processing capabilities [19]. The ML models utilizing electronic health records (EHRs), which show promise in outperforming traditional risk scores like QRISK3 and atherosclerotic cardiovascular disease (ASCVD) [20]. Supervised ML builds predictive algorithms from labeled data, aiding in outcome prediction and image classification [21]. ML and deep learning (DL) also improve myocardial segmentation and quantification by reducing operator variability [22]. FDA-approved AI tools such as EchoGo Heart Failure 2.0, HeartKey® Rhythm, Tempus, ECG-AF, Corvair, and ELEFT exemplify recent advancements in AI-driven CVD diagnostics [8].

AI in CV diagnostics: The AI-powered tools are enhancing traditional diagnostic methods by providing rapid and accurate interpretation of CV imaging and electrophysiological data. The DL models are being employed in echocardiography, computed tomography (CT), and magnetic resonance imaging (MRI) to detect conditions such as coronary artery disease (CAD), cardiomyopathies, and valvular heart disorders with high precision. The AI-assisted electrocardiogram (ECG) interpretation can identify subtle patterns indicative of arrhythmias, myocardial infarction (MI), and HF at an early stage [23]. The DL algorithms provide an improved image, which is at the risk of image deterioration and motion artefacts during CT. Meanwhile, ML detects wall motion abnormalities, which are equivalent to trained echocardiographers and superior to trainees [24]. Additionally, the ML algorithms have shown strong accuracy in coronary artery calcium scoring, offering scalable solutions to detect undiagnosed CAD and improve healthcare efficiency [25]. 

Wearable technology and remote monitoring: The integration of wearable sensors and mobile health applications enables continuous monitoring of vital parameters such as heart rate, blood pressure, and oxygen saturation. Smartwatches and biosensors equipped with AI-based analytics can detect atrial fibrillation, monitor HF progression, and provide alerts for abnormal CV events. These innovations allow for proactive interventions and reduce hospital readmissions [26]. Although AI/ML applications using mobile device data offer significant potential, they raise specific ethical concerns. Since the data are owned by patients, it is essential to ensure privacy, data integrity, and interoperability among all stakeholders [5].

AI-driven decision support systems: Clinical decision support systems leveraging AI are aiding healthcare providers in risk stratification, treatment planning, and personalized patient management [27]. The AI models analyzed electronic health records (EHRs) and large-scale datasets, can predict adverse CV events, guide anticoagulation therapy, and optimize medication regimens based on individual patient profiles [28].

Digital therapeutics and telemedicine: Digital therapeutics, including AI-driven mobile applications, provide patients with personalized guidance on medication adherence, lifestyle modifications, and rehabilitation programs [29]. Telemedicine platforms powered by AI facilitate virtual consultations, remote patient monitoring, and digital coaching, ensuring continuity of care for individuals with CV conditions, especially in remote or underserved regions [30]. A study by Gray et al. reported that digital technology with clinical data recording, along with clinician feedback and structured follow-up, provides efficacious outcomes and reduces hospital readmissions [31].

Limitations and challenges of AI: Limitations of AI include poor data quality, biased algorithms, patient mistrust in the healthcare system, inadequate clinician education, and high cost and care inaccessibility [32]. Technological challenges include data integration, infrastructure requirements, data quality and consistency, real‐time processing, ethical and regulatory challenges such as data privacy and security, informed consent for the use of personal data and information, algorithm fairness and biases, safety and transparency [22]. The ML models are unable to detect time-to-event variations and censored patients, which limits their performance remarkably. When the ML models rely on a high number of variables and interactions for risk prediction (e.g., black box model), identifying specific therapeutic targets can be challenging or impossible [21]. Some AI/ML algorithms work well for different clinical scenarios, while some may not. For AI/ML algorithm training, accurate ground-truth labels are essential. However, tools like natural language processing for rapidly generating labels may cause errors [5]. 

Innovations in therapeutics

1. Regenerative and Stem Cell Therapies in CV Care

Regenerative medicine and stem cell therapies have emerged as promising approaches for repairing and regenerating damaged cardiac tissue. These therapies aim to restore myocardial function in patients with HF, MI, and other CV disorders by leveraging the regenerative potential of stem cells and biomaterials [33].

Stem cell-based cardiac regeneration: Various types of stem cells, including mesenchymal stem cells (MSCs), induced pluripotent stem cells (iPSCs), and cardiac progenitor cells, have been investigated for their ability to regenerate heart tissue. Clinical trials have demonstrated that stem cell transplantation can improve cardiac function by promoting neovascularization, reducing fibrosis, and enhancing cardiomyocyte survival [34].

Tissue engineering and 3D bioprinting: Advancements in tissue engineering and 3D bioprinting are enabling the development of bioengineered heart tissues and patches for myocardial repair. These technologies facilitate the creation of functional cardiac constructs using patient-derived cells, offering a personalized approach to cardiac regeneration [35].

Gene and cell therapy combinations: Emerging strategies involve combining gene therapy with stem cell transplantation to enhance cardiac repair mechanisms. Genetic modifications of stem cells, such as overexpressing angiogenic factors or anti-apoptotic genes, have shown potential in improving cell survival and therapeutic efficacy in preclinical studies [36]. Despite the promise of regenerative therapies, challenges such as immune rejection, cell retention, and long-term efficacy remain areas of ongoing research. Further clinical trials and advancements in bioengineering will be crucial in translating these therapies into widespread clinical practice.

Challenges in regenerative and stem cell therapies: The iPSCs and MSCs face major key challenges such as tumorigenicity, genetic instability, unpredictable differentiation, immune rejection, difficulties in standardization, scalability, and integration. Although the MSCs are safer and less tumorigenic than iPSCs, they face challenges such as limited proliferation, senescence, and population heterogeneity, which may affect the therapeutic consistency. Strict validation, standardization, and regulatory oversight to ensure clinical safety and efficacy are essential in both MSCs and iPSCs. Despite these challenges, iPSC-based therapies are promising due to their expandability, potential for immune compatibility, and pluripotency [37].

2. Next-Generation Pharmacotherapies

The landscape of CV pharmacotherapy is evolving with the introduction of novel drug classes and targeted therapies that offer enhanced efficacy and safety profiles. Sodium-glucose cotransporter-2 (SGLT2) Inhibitors and glucagon-like peptide-1 (GLP-1) receptor agonists have demonstrated significant CV benefits beyond glucose control. These drugs reduce HF hospitalizations, improve renal outcomes, and lower CV mortality in patients with diabetes and heart disease [38].

Proprotein convertase subtilisin/kexin type 9 (PCSK9) inhibitors and lipid-lowering therapies: PCSK9 inhibitors, such as evolocumab and alirocumab, offer potent LDL cholesterol reduction, particularly in high-risk patients [39]. Novel small interfering RNA (siRNA)-based therapies, like inclisiran, provide sustained lipid-lowering effects with less frequent dosing [40].

Gene Therapy for CVD Advancements in gene therapy are enabling the development of targeted interventions for inherited lipid disorders, HF, and atherosclerosis. Experimental therapies involving CRISPR-based gene editing and viral vector-mediated gene delivery hold promise for long-term disease modification [41]. Adeno-associated virus (AAV) and recombinant AAV gene therapies are the leading vectors in cell and gene therapy, offering new opportunities for management of inherited cardiomyopathies and HF [42,43]. The limitations of gene therapies mainly included inadequate delivery of the genetic material to achieve therapeutic levels, the potential for immune responses (humoral or cellular) with repeated administration, and limited or short-lived gene expression. The AAV vectors can cause systemic leakage of the gene transfer and provoke immune responses. Additionally, systemic leakage of angiogenic gene factors may lead to harmful off-target effects. The diseased vessel wall also poses a barrier for effective gene delivery when injected into blood vessels, resulting in low transgene efficiency and poor clinical outcomes [44].

3. Advances in Interventional Cardiology

Minimally invasive procedures, such as transcatheter aortic valve replacement (TAVR), percutaneous coronary interventions, and catheter-based ablation therapies, have significantly improved outcomes for patients with structural and ischemic heart disease. Innovations in bioresorbable stents, drug-coated balloons, and robotic-assisted interventions are further enhancing procedural success rates and reducing complications [45]. Early initiation of invasive therapy improves durable survival as well as improves long-term survival and reduces late MI and rehospitalization in individuals [46,47].

Regenerative therapies using stem cells, tissue engineering, and gene editing offer promising outcomes for the management of CVD, although immune rejection and cell variability are some of the major challenges that still persist. Next-generation pharmacotherapies, including SGLT2 inhibitors, PCSK9 inhibitors, and gene therapies, also contribute to CVD management. In interventional cardiology, established procedures like TAVR are now routine practice among cardiologists, while emerging technologies such as robotic-assisted interventions are still under clinical development. Future therapeutic strategies may integrate regenerative medicine, gene therapy, and pharmacological advances for more comprehensive and personalized CV care.

Preventive strategies and public health impact

1. Lifestyle Modifications and Digital Health Interventions

Lifestyle modifications remain one of the most effective strategies for preventing and managing CVD. Digital health interventions have significantly enhanced the ability to monitor, encourage, and sustain these modifications. Adopting a heart-healthy diet, such as the Mediterranean or DASH (Dietary Approaches to Stop Hypertension) diet, plays a crucial role in CV risk reduction [48].

Regular physical activity is essential for CV health. Wearable fitness trackers, mobile health applications, and virtual coaching platforms help patients adhere to exercise regimens tailored to their fitness levels and health conditions. Gamification elements in apps enhance motivation and long-term adherence. Psychosocial stress is a well-recognized risk factor for CVD. Digital mental health interventions, including mindfulness and meditation apps, offer guided relaxation exercises, stress tracking, and behavioral therapy programs to improve psychological well-being and reduce CV risk [48]. Smoking and excessive alcohol consumption significantly contribute to CV morbidity. Digital tools, including AI-driven nutrition apps, track food intake, provide personalized dietary recommendations, and guide patients in making healthier choices [48]. In recent years, smartphone use has increased tremendously in tracking food consumption and calculating nutritional value. Smartphones have played a key role in accessing food analytical techniques for on-site analysis as a readily available and convenient integrated interface, connectivity, and remote sensing platforms [49]. Several AI-driven nutrition apps exemplify this trend, including Calorie Counter-MyNetDiary, MyFitnessPal, Lose It!-Calorie Counter for Diet & Weight Loss, and Lifesum-Diet Plan, Macro Calculator & Food Diary, etc. [50].

Digital health interventions, including smartphone applications, online support communities, and AI-driven behavioral coaching, have demonstrated effectiveness in smoking cessation and reducing harmful alcohol use. Digital coaching, reminders, and automated feedback systems enhance patient engagement and adherence to CV health strategies.

2. Population-Level Screening and Risk Prediction Models

Early identification of individuals at high risk for CVD is essential for timely intervention and prevention. Population-level screening programs and risk prediction models have become indispensable tools in CV care, enabling stratification of at-risk individuals and guiding evidence-based prevention strategies. Population-level screening aims to identify asymptomatic individuals at increased risk of developing CVD. Screening strategies vary based on age, sex, ethnicity, and other risk factors. Traditional screening approaches include blood pressure measurement, lipid profiling, fasting glucose levels, and electrocardiography [51].

Risk prediction models estimate an individual's likelihood of developing CVD over a defined period. Traditional models include Framingham Risk Score (FRS), one of the earliest models, which assesses the 10-year risk of coronary heart disease based on age, sex, cholesterol levels, blood pressure, smoking status, and diabetes. Reynolds Risk Score (RRS), incorporates high-sensitivity C-reactive protein (hs-CRP) to refine risk assessment, particularly in women. The ASCVD Risk Calculator, developed by the American College of Cardiology/American Heart Association (ACC/AHA), estimates 10-year and lifetime risks of atherosclerotic cardiovascular disease [52]. Studies comparing various risk calculators suggested that the Framingham Risk Score for CVD has demonstrated superior performance in identifying high-risk patients [53,54]. The FRS and ASCVD risk scores are effective risk-predicting tools for CVD in an Indian population despite being Western-developed risk scores. A study by Duttagupta et al. reported that the ASCVD risk calculator exhibited higher discriminative ability for predicting severe CAD [55]. 

Advancements in risk prediction integrate novel biomarkers and machine learning algorithms to enhance accuracy. AI-driven risk models analysed large datasets from electronic health records, wearable devices, and genomic data to provide individualized risk assessments. Polygenic risk scores (PRS) leverage genome-wide association studies (GWAS) to refine CV risk stratification [54].

Emerging modalities incorporate genetic testing, biomarker analysis, and advanced imaging techniques such as coronary artery calcium (CAC) scoring and carotid intima-media thickness (CIMT) assessment [51]. Role of imaging and biomarkers in risk prediction: Advanced imaging techniques have improved CVD risk assessment beyond traditional models. Coronary artery calcium (CAC) scoring: Provides a direct measure of subclinical atherosclerosis and enhances risk stratification, especially in intermediate-risk individuals. Carotid Intima-Media Thickness (CIMT): Evaluates atherosclerotic burden and predicts future CV events. Cardiac MRI and CT Angiography: Offer detailed structural and functional cardiac assessments, aiding in early disease detection. Emerging biomarkers, such as high-sensitivity troponins, pro-B-type natriuretic peptides (BNP/NT-proBNP), and inflammatory markers, further refine risk assessment and guide therapeutic decisions [56].

Recent studies have demonstrated the superior performance of AI models compared to traditional risk scores in predicting clinical outcomes. A systematic review and meta-analysis of 558,233 HF patients compared machine learning models with traditional risk scores for predicting all-cause mortality and readmission reported higher summary C-statistic of the top-performing ML models for all-cause mortality (mean difference (MD): 0.76; 95% CI: 0.72 to 0.80), compared to traditional risk scores (MD: 0.71; 95% CI: 0.68 to 0.74). The difference in C-statistic between ML models and traditional methods was 0.05 (95% CI: 0.04-0.06; P=0.05) [57]. Similarly, another systematic review and meta-analysis reported that AI models have a higher predictive accuracy as compared to traditional risk scores in predicting post-TAVI mortality [58].

Challenges and future directions

Despite these advancements, challenges such as accessibility, cost-effectiveness, and ethical considerations must be addressed. Ensuring equitable access to innovative therapies and integrating emerging technologies into routine practice requires collaborative efforts between researchers, clinicians, and policymakers [59]. Despite significant advancements in CV research and clinical practice, several challenges remain. Addressing these hurdles is critical for ensuring equitable access, improving patient outcomes, and advancing the next generation of CV care.

1. Disparities in Access to Care

One of the biggest challenges in CV care is the inequitable access to diagnostic tools, advanced therapies, and preventive interventions. Socioeconomic factors, geographic limitations, and healthcare infrastructure disparities contribute to variations in patient outcomes. Developing countries and rural populations often lack access to cutting-edge diagnostics, specialist care, and life-saving interventions. Policymakers and healthcare systems must focus on improving healthcare access through telemedicine, mobile health clinics, and cost-effective screening programs. Expanding health insurance coverage and investing in primary care infrastructure can help bridge these gaps [60].

2. Overdiagnosis and Overtreatment

The increased availability of high-resolution imaging, genetic testing, and AI-driven diagnostics has led to concerns about overdiagnosis and overtreatment. Some patients may undergo unnecessary procedures, excessive pharmacotherapy, or lifestyle modifications based on marginal risk assessments, leading to increased healthcare costs and potential adverse effects [61]. Overdiagnosis causes unnecessary psychological suffering of people and leads to overtreatment, which can harm patients and divert resources from patients most in need or severely ill [62]. Risk stratification tools should be refined to ensure that screening and treatment interventions are targeted at high-risk individuals. Guidelines should be regularly updated based on real-world evidence to minimize unnecessary interventions [63].

3. Data Privacy, Security, and Integration

The expansion of AI, digital health technologies, and big data analytics in CV care raises significant concerns about data privacy, security, and interoperability. Many healthcare systems lack standardized methods for securely integrating electronic health records (EHRs), wearable device data, and genomic information, making it challenging to leverage these resources effectively. Governments and regulatory bodies must establish robust data protection laws and interoperability standards to facilitate secure data sharing among healthcare providers. Blockchain technology and federated learning models (such as the TabNet model) may enhance data security while maintaining patient confidentiality [63,64].

4. Implementation of AI and Digital Health in Clinical Practice

Although AI-driven tools and digital health solutions have demonstrated immense potential, their widespread adoption in clinical settings remains slow. Barriers include the need for physician training, regulatory approvals, and integration into existing clinical workflows. Patients' attitudes, like agreeableness, openness, and conscientiousness, toward AI application usage in the management of disease may act as a barrier [65]. Hence, AI-based tools must be developed with user-friendly interfaces, and healthcare professionals should receive training on their use. Regulatory agencies should establish clear guidelines for AI implementation to ensure safety and efficacy [17].

5. Personalized Medicine and Its Challenges

While precision medicine and genomics hold great promise, their implementation is complex. Genetic testing and multi-omics approaches require sophisticated infrastructure, specialized expertise, and significant financial investment [66]. Various gene-like single-nucleotide polymorphisms (SNPs) potentially contribute toward an increased risk of coronary heart disease. The SNPs are the most common sources of genetic variations in CVD patients [67]. Additionally, the interpretation of genetic findings and their clinical relevance can be challenging. Advances in bioinformatics and machine learning can enhance the interpretation of genetic data, making precision medicine more accessible [66]. The regulatory framework in the medical device’s major markets, like Europe, the USA, and Japan, differs significantly. Collaborations between these markets are essential to ensure compliance with local requirements, due to region-specific rules governing product approval, safety, and quality [68]. Additionally, collaborations between academic institutions, pharmaceutical companies, and healthcare systems can drive the development of cost-effective precision medicine solutions [66].

6. Addressing CVD in an Aging Population

As global life expectancy increases, the prevalence of age-related CVD, such as HF, atrial fibrillation, and atherosclerosis, continues to rise. Managing multimorbidity and polypharmacy in older adults poses a significant challenge. Research should focus on age-specific treatment strategies, frailty assessment tools, and holistic care approaches to optimize CV management in older adults. Digital health interventions can aid in remote monitoring and adherence to treatment plans [69]. 

7. The Need for Real-World Evidence (RWE) in CV Care

Many clinical trials focus on selected patient populations, limiting the generalizability of findings to real-world settings. There is a growing need for real-world evidence to assess the long-term safety, efficacy, and cost-effectiveness of novel CV therapies. Expanding real-world evidence studies, registries, and post-marketing surveillance programs will provide insights into the effectiveness of new treatments in diverse populations. AI-driven analytics can enhance the utility of real-world data for clinical decision-making [70].

## Conclusions

The future of CV care lies in the seamless translation of research innovations into clinical practice. The integration of AI along with precision medicine enables medical professionals and patients to access highly accurate diagnostic and treatment outcomes. With continued advancements in precision medicine, AI-driven diagnostics, and novel therapeutics, the paradigm of CV management is shifting towards a more personalized, proactive, and patient-centred approach. By adopting this strategy, CVD management can witness significant progress, resulting in improved CV health outcomes. Ongoing research and interdisciplinary collaboration will be key in shaping the next era of CV care.
